# Spatiotemporal Context Awareness for Urban Traffic Modeling and Prediction: Sparse Representation Based Variable Selection

**DOI:** 10.1371/journal.pone.0141223

**Published:** 2015-10-23

**Authors:** Su Yang, Shixiong Shi, Xiaobing Hu, Minjie Wang

**Affiliations:** Shanghai Key Laboratory of Intelligent Information Processing, School of Computer Science, Fudan University, Shanghai, China; Beihang University, CHINA

## Abstract

Spatial-temporal correlations among the data play an important role in traffic flow prediction. Correspondingly, traffic modeling and prediction based on big data analytics emerges due to the city-scale interactions among traffic flows. A new methodology based on sparse representation is proposed to reveal the spatial-temporal dependencies among traffic flows so as to simplify the correlations among traffic data for the prediction task at a given sensor. Three important findings are observed in the experiments: (1) Only traffic flows immediately prior to the present time affect the formation of current traffic flows, which implies the possibility to reduce the traditional high-order predictors into an 1-order model. (2) The spatial context relevant to a given prediction task is more complex than what is assumed to exist locally and can spread out to the whole city. (3) The spatial context varies with the target sensor undergoing prediction and enlarges with the increment of time lag for prediction. Because the scope of human mobility is subject to travel time, identifying the varying spatial context against time lag is crucial for prediction. Since sparse representation can capture the varying spatial context to adapt to the prediction task, it outperforms the traditional methods the inputs of which are confined as the data from a fixed number of nearby sensors. As the spatial-temporal context for any prediction task is fully detected from the traffic data in an automated manner, where no additional information regarding network topology is needed, it has good scalability to be applicable to large-scale networks.

## Introduction

Short-term traffic flow prediction plays an important role in intelligent transportation in terms of both route planning and traffic management. The early efforts are focused on predicting traffic time series at a single point. Season ARIMA model [1] is the most widely used parametric model. The nearest neighbor method in terms of pattern recognition is an alternative solution [2]. Besides, the state-space model enables traffic flow prediction to be solved in the framework of control theory [3]. Neural networks are also representative models for traffic flow prediction [4][5]. Support vector regression is yet well known in the context of traffic flow prediction [6].

However, the rising of big data analytics brings in a new chance to revisit city dynamics from a novel point of view due to the massive data of human mobility available from taxi GPS traces [7][8][9], locations inferred from mobile phone positioning [10], web logs with geotags [11], and measurements from traditional traffic meters like loop detectors [12]. Recently, it is realized that traffic flows are coupled on a road network via mutual interactions such that the evolution of the traffic flow at each site is not independent to but constrained by those of the others at the whole city scale [12][13]. Correspondingly, the recent trend is shifted to making use of spatial-temporal correlations among observations at multiples sites to improve prediction. In [14][15], vector ARMA model [16] incorporating both spatial and temporal correlations is applied to predict the traffic flow at one site. In [17], sparse representation is applied to optimize the solution. The standpoint of these methods is: Traffic flows will affect each other in a nearby region and traffic jams will propagate from one place to the nearby roads [18]. Hence, the data from a couple of nearby sensors are incorporated into the input to the predictor [14][15][19][20][21][22] with such an assumption that the evolution of the traffic flow at a given site is only subject to the states of its neighbors.

Due to the lack of the understanding about how far traffic flows affect each other, yet, the correlated sensors are determined empirically for these methods. The common practice is: The number of the manually selected sensors for any prediction task remains fixed, usually below 25, regardless the time lag for prediction and the location specific of the site undergoing prediction. For example, only 25, 15, 10, and less than 10 nearby sensors are considered as the spatial contexts to affect the prediction at a given site in [14][15][18][23], respectively. For spatiotemporal data, however, simply incorporating spatially distributed sensors and attributes may actually generate predictions that are worse than non-spatial models unless the data from the selected sensors do truly contribute to the predicted value [19]. In [15], it is showed that injudicious use of data from neighboring locations may actually decrease prediction accuracy since using relatively few, dispersed sensors fails to account for the spatial dependencies while the prediction accuracy may improve significantly if spatiotemporal relationships are captured by increasing the number of sensors [19]. Since the manual work to determine spatial correlations will become unaffordable if scaled to the whole network level, the timely issue for traffic flow prediction is to develop an approach that can detect the spatial context automatically. So far, how to infer such global context to enable better prediction remains an open problem yet.

In addition, traffic flows are assumed to have a long-term temporal dependency, which means that the formation of the present traffic flow is subject to the traffic flows of a long-term course in the past. Based on such assumption, the existing works employ high-order prediction models to capture the temporal correlation among traffic flows [14][15]. Yet, such an assumption about long-term temporal dependency has never been checked to date.

This study contributes to explore the spatiotemporal correlation problem from the following perspectives: (1) We discover the spatiotemporal correlations among the traffic data in the framework of sparse representation since it enables to decompose the outcome of the prediction into the linear combination of as few as possible relevant variables. (2) We deploy a high-order dynamic model to investigate how far the current traffic flow is affected by the traffic flows of a couple of time steps ago.

We test the proposed method based on the real-world data achieved from 3254 loop detectors on Twin Cities Metro freeways. The experiments show that the proposed method can reach as high as 89.68% accuracy at the average level for traffic flow prediction based on only 100–500 relevant sensors selected through sparse representation as the input, which outperforms remarkably the least squared fitting method and the methods by confining the spatial context into just a certain range of the neighborhood. Moreover, we observed some interesting laws regarding how the spatial contexts to predict traffic flows are shaped, which have never been reported before from a big data point of view: (1) The prediction performance of high-order models is nearly equal to that of 1-order model, which means that the current traffic flow is mainly shaped by the traffic flows of the last time step. (2) By inferring the minimum number of variables/sensors to reconstruct the traffic flow at the sensor of interest by means of spares representation, we obtain a global spatial context for most prediction tasks. In general, hundreds of sensors distributed on the whole road network, not just a local region, are relevant to a prediction task [24], which implies a much wider range spatial context than just a couple of neighboring sensors as assumed previously. This further evidences the view of [12]. (3) The number of relevant sensors is subject to the targeted sensor undergoing prediction due to the location-specific topology in the road network. Besides, the spatial context enlarges with the increment of the time lag for prediction while the performance degradation is limited due to the fact that a longer traveling distance allows travelers to visit a larger area to affect the traffic flows there [24]. This means that the extent of spatial correlation corresponding with the scope of human mobility is subject to travel time and identification of the varying spatial context against time lag is crucial for prediction. Compared with the existing prediction methods the spatial context of which are confined within a fixed number of sensors in the neighborhood, the global spatial context turned out from sparse representation promises better prediction performance due to the more practical and adaptive spatial context subject to the sensor undergoing prediction as well as the time delay.

## Related Works

There are two modalities of data to deal with traffic modeling and prediction. The first one is the assembly of a large number of digital traces of moving objects such as taxi GPS traces and mobile phone positions. This category of data record the whole journey of each individual to form explicit origin-destination flows but such digital traces compose only a small subset of the whole collection of moving objects in a city. The other category of data is obtained from the traditional traffic flow meters mounted at fixed positions like loop detectors, where the traveling route of an individual is not available but the travel behaviors covering densely the whole city can be observed. Here, we use the latter one to conduct our research since such category of data distribute ubiquitously in the whole city and available at any time, which fits well into the goal of mining the global spatiotemporal context for traffic modeling and prediction.

Traffic flow prediction is a classical problem in the literature of transportation engineering. The recent trend is shifted to spatiotemporal correlation based prediction so as to foresee the traffic time series at one site from the mutual interactions among the relevant traffic flows. However, the state-of-the art prediction models rely on the input from the nearby region in which the number of correlated sensors is up to 25 sensors [14][15][19][20][21][22]. In [14], spatiotemporal correlations are determined empirically but the labor-intensive works as such are not applicable to large-scale networks. In [15], it is showed that injudicious use of data from neighboring locations may actually decrease prediction accuracy since using relatively few, dispersed sensors fails to account for the spatial dependencies while the prediction accuracy may improve significantly if spatiotemporal relationships are captured by increasing the number of sensors [19]. In [12], the experiments based on the traffic data achieved from thousands of sensors in Twin cities (a city in US) for one year show that the number of the sensors relevant to the prediction task at a given site is in general over 100, which indicates a global context for modeling and predicting the traffic flow at a given site. Shortly, it is further confirmed in the sense of complex network based experiments that traffic fluctuations in London are correlated over the whole city [13]. This means that we have to reconsider the problem of spatiotemporal correlations among traffic data from a big data point of view for the sake of prediction. Except [12], however, almost all the other works try to solve the spatiotemporal context from the nearby region around the site undergoing prediction. In [20], a variable selection method based on repeatedly random selection of subspaces along with Gaussian Mixture Models is proposed for traffic flow prediction but it is only applied to a small scale network due to the time consuming repeated selection of subspaces as well as the training of Gaussian Mixture Models. In [21], a variable selection method aiming to reduce the size of neural networks is proposed for traffic flow prediction. Yet, this method is only applicable to neural networks and the experiment is conducted on a local region with 7 sensors. In [22], variable selection is employed to reduce the fuzzy rules into a concise set. However, the variable selection is performed on the sensors on an identical road only, not the whole network, and the method is merely applicable to fuzzy system based congestion prediction. The aforementioned works are ad hoc in that if a variable selection method can only work for a specific predictive model the input of which is focused on a local region, the scalability would be limited. Although [12] is an exceptional work, where feature selection is proposed to identify relevant sensors for traffic congestion prediction at the whole city scale, but the performance is not satisfactory and the method is not applicable to traffic flow prediction, which is different from traffic congestion prediction. In [25][26], feature learning and traffic flow prediction are organized in a pipelined procedure in terms of deep learning. Due to the complex neural structures, however, the spatial dependences among traffic flows are not explicitly visible, nor explainable.

Consistent with [12][13], for traffic flow prediction, the role of global spatial contexts should be explored. In this study, we propose to make use of sparse representation technique as a variable selection method [27] in exploring the spatial correlations among the traffic data of the whole city. The goal of sparse representation is to obtain as small as possible fitting error with as few as possible variables by forcing most fitting coefficients in a predictor to be zeros through an optimization procedure.

In view of the literature, the uniqueness of this work can be summarized as follows: (1) We propose to solve the spatiotemporal context mining problem for traffic flow prediction in a unified framework via sparse representation. By applying a high-order model other than the 1-order model in [24], we discover not only spatial context but also temporal context simultaneously. Moreover, it is a generic variable selection method applicable to a variety of predictors such as vector ARMA models and neural networks, which are the dominant models in terms of traffic flow prediction. (2) Other than most existing works, it is a big data analytics scheme. By mining the correlations among the traffic data from the whole city, some results disagreeing with traditional studies are observed experimentally. Although sparse representation has been widely used in a variety of engineering applications, even in traffic flow prediction [17], where the spatial context is confined to be less than 20 sensors in a local region only, to the best of our knowledge, the proposed scheme is a new solution to identify spatiotemporal contexts from the big data of the whole city for traffic flow prediction.

## Traffic Modeling and Prediction based on Sparse Representation

In the following, we first introduce the high-order dynamic model for traffic flow prediction and then present the sparse representation based solution to this model.

### Vector Autoregressive Model as Predictor

A predictor based on Vector Autoregressive (VAR) model [15] of order *p* can be written in the form of

$$\begin{array}{c} \;v_{t}=W_{1}v_{t-1}+W_{2}v_{t-2}+...+W_{p}v_{t-p}+u_{t}\; \end{array}$$ (1)

where $v_{t}={\left[v_{t}^{1},v_{t}^{2},\ldots v_{t}^{i},\ldots ,v_{t}^{m}\right]}^{T}$ represent the traffic volume data sampled at time *t* from all the *m* sensors in the road network, namely the state of network flow at time *t*, *i* the index of the *i*th sensor, and $v_{t}^{i}$ the traffic volume value of the *i*th sensor recorded at time *t*. The goal is to compute the predicted network state *v*_*t*_ from the linear combination of the weighted states at previous 1 to *p* time steps. Here, *W*_*k*_ is a matrix containing the corresponding coefficients to weight the contribution of *v*_*t*−*k*_ in predicting *v*_*t*_, which is in the form of

$$W_{k}=\;\left[\begin{array}{c} W_{k}^{1}\\ W_{k}^{2}\\ \vdots \\ W_{k}^{m} \end{array}\right]\;=\;\left[\begin{array}{cccc} w_{1,k}^{1} & w_{2,k}^{1} & \cdots & w_{m,k}^{1}\\ w_{1,k}^{2} & w_{2,k}^{2} & \cdots & w_{m,k}^{2}\\ \vdots & \vdots & & \vdots \\ w_{1,k}^{m} & w_{2,k}^{m} & \cdots & w_{m,k}^{m} \end{array}\right]$$ (2)

where $w_{i,k}^{j}$ represents the correlation degree between sensor *j* and sensor *i* in terms of traffic flow prediction at sensor *j* with time lag *k* and $W_{k}^{j}=[w_{1,k}^{j},w_{2,k}^{j},...w_{m,k}^{j}]$ is a row vector to weight the contributions of all the *m* sensors in predicting the traffic volume at sensor *j*. $u_{t}={\left[u_{t}^{1},u_{t}^{2},\ldots ,u_{t}^{m}\right]}^{T}$ is a *m*-dimensional vector representing independently and identically distributed random noises. According to Eq (1), the predictor of order *p* corresponds with that the traffic volume values of from 1 step to *p* time steps ahead are applied as input in predicting the present traffic volume. In accordance with Eq (1), we can easily reach the prediction at sensor *j*, that is,

$$\begin{array}{c} \;v_{t}^{j}=W_{1}^{j}v_{t-1}+W_{2}^{j}v_{t-2}+...+W_{p}^{j}v_{t-p}+u_{t}^{j}=\sum_{k=1}^{p}\sum_{i=1}^{m}w_{i,k}^{j}v_{t-k}^{i}+u_{t}^{j}\; \end{array}$$ (3)

In view of Eq (3), the observation at sensor *j* at time step *t*, say $v_{t}^{j}$, is modeled as a linear combination of from 1-step-ahead to *p*-step-ahead observations from all sensors plus an additional term representing random noise. Here, $w_{i,k}^{j}$ functions to weight the spatial-temporal correlation between sensor *j* and sensor *i* with *k*-step time lag. Note that in Eq (1), the nonzero elements in $W_{k}^{j}=[w_{1,k}^{j},w_{2,k}^{j},...w_{m,k}^{j}]$ reflect the spatial correlations between the data from the corresponding sensors and the predicted traffic volume $v_{t}^{j}$ and $W_{1}^{j},W_{2}^{j},...,W_{p}^{j}$ reflect the dynamic nature of the evolution of the traffic flow, that is, how long time the effect of the previous states will last to affect the present state, which is also referred to as temporal correlation. If only $W_{1}^{j}$ contains nonzero elements, it means that the present state of the network flow is only dependent on the network state at the immediately previous step, say, Markov property. Once $w_{i,k}^{j}=0$, it means that the observations obtained at sensor *i* of *k* steps ahead do not contribute to the prediction task at sensor *j*. Intuitively, the number of the sensors relevant to the prediction task at a given sensor is limited, which means that the number of nonzero weights in the predictor should also be limited. However, if we use least square fitting or maximum Likelihood method to estimate the coefficients, all the VAR coefficients could be nonzero in practice. So, we apply sparse representation to solve the VAR model to find out the minimum number of variables truly contributive to the prediction task. Prior to performing sparse representation based optimization, we have to reformulate the VAR model into a form to which sparse representation is applicable. By means of that, the spatial and temporal correlation can be solved simultaneously in a simple equation. The details are described below.

### Solving VAR Model via Sparse Representation

Let the weights in Eq (3) be reformatted as follows:

$$\begin{array}{l} w^{j}={\left[W_{1}^{j},W_{2}^{j},\ldots ,W_{p}^{j}\right]}^{T}\\ \;={\left[w_{1,1}^{j},w_{2,1}^{j},\ldots ,w_{m,1}^{j},w_{1,2}^{j},w_{2,2}^{j}\ldots ,w_{m,2}^{j},\ldots ,w_{1,p}^{j},w_{2,p}^{j}\ldots ,w_{m,p}^{j}\right]}^{T} \end{array}$$ (4)

Correspondingly, we reorganize the traffic volume data observed at all sensors at the (*t*-1) time step to the (*t*-*p*) time step as

$$\begin{array}{l} V_{t-1}=[{\left(v_{t-1}\right)}^{T},{\left(v_{t-2}\right)}^{T},\ldots {\left(v_{t-p}\right)}^{T}]\\ \;=[v_{t-1}^{1},v_{t-1}^{2},\ldots ,v_{t-1}^{m},v_{t-2}^{1},v_{t-2}^{2}\ldots ,v_{t-2}^{m},\ldots ,v_{t-p}^{1},t_{t-p}^{2}\ldots ,v_{t-p}^{m}] \end{array}$$ (5)

Then, we can rewrite Eq (3) in another form as follows:

$$\begin{array}{c} \;v_{t}^{j}=V_{t-1}w^{j}+u_{t}^{j}\; \end{array}$$ (6)

Prior to prediction, it is necessary to learn the parameter *w* ^*j*^ from historical data. Let $v^{j}={\left[v_{\tau +1}^{j},v_{\tau +2}^{j},\ldots ,v_{\tau +n}^{j}\right]}^{T}$ and $V={\left[V_{\tau }^{T},V_{\tau +1}^{T},\ldots ,V_{\tau +n-1}^{T}\right]}^{T}$, where *v* ^*j*^ is the time sequence acquired at sensor *j* and *V* is a matrix with *n* rows and *m* × *p* columns. In accordance with Eq (6), the training task in the least-square sense is to adjust *w* ^*j*^ so as to minimize

$$\begin{array}{c} \;J=\left|\right|v^{j}-Vw^{j}{\left|\right|}_{2}^{2}\; \end{array}$$ (7)

However, sparse representation based solution gains advantage over least squared fitting in that only a few elements in *w* ^*j*^ have nonzero values, which can reveal the spatially and temporally correlated components in *w* ^*j*^ that are contributive to the prediction. The sparse representation based solution of the VAR model can be formulated as follows:

$${\hat{w}}^{j}=\text{arg}\underset{w^{j}}{\text{min}}{\left\|w^{j}\right\|}_{0}\;subject\;to\;|Vw^{j}-v^{j}|{|}_{2}^{2}⩽\varepsilon_{0}$$ (8)

where ${\left\|w^{j}\right\|}_{0}$ is the *l* ^0^-norm of *w* ^*j*^, corresponding with the number of nonzero elements in vector *w* ^*j*^, and *ɛ*_0_ is a parameter to control the fitting error to be small. In general, solving directly Eq (8) proves to be an NP-hard problem. Thus, a couple of efficient pursuit algorithms have been proposed to search approximated solutions. The simplest ones are *matching pursuit* (MP) and *orthogonal matching pursuit* (OMP) algorithms, which use the so-called greedy strategy trying to obtain globally optimal solution from a couple of local optimizations. A second well-known pursuit approach is the *Basis Pursuit* (BP). It replaces the *l* ^0^-norm ${\left\|w^{j}\right\|}_{0}$ in Eq (8) with a *l* ^1^-norm ${\left\|w^{j}\right\|}_{1}$, where ${\left\|w^{j}\right\|}_{1}$ is the sum of the absolute values of all the elements in *w* ^*j*^. Chen et al. prove that *l* ^1^-norm solution has indeed sparse property [28] and Elad verifies experimentally that such *l* ^1^-norm solution can approach the true sparse solution [29]. By applying the *l* ^1^-norm and Lagrange multiplier *λ* to Eq (8), the constrained optimization problem can be converted into the following unconstrained one:

$${\hat{w}}^{j}=\text{arg}\underset{w^{j}}{\text{min}}\left\{\lambda \;\|w^{j}{\|}_{1}+\frac{1}{2}\|\;Vw^{j}-v^{j}|{|}_{2}^{2}\right\}$$ (9)

To solve Eq (9), we apply the well-known least angle regression stagewise (LARS) algorithm [30], by means of which a solution with small fitting error can be obtained efficiently. Here, the open-source software SPAMS (http://spams-devel.gforge.inria.fr/-index.html) can be used to obtain the solution. Once the training procedure as described above is finished, we can perform the prediction via

$$\begin{array}{c} \;{\bar{v}}^{j}=V{\hat{w}}^{j}\; \end{array}$$ (10)

where ${\bar{v}}^{j}$ is the estimated traffic volume values in correspondence with *v* ^*j*^. The details of the sparse representation method can be found in [29].

## Experiments Findings

We first describe the experimental settings. Then, we present the results of temporal correlation mining and spatial context mining to justify that temporal correlation can be simplified as 1-order dynamic model while spatial context can spread widely up to the edge of a city. Finally, we figure out how spatial context varies with the senor of interest as well as time delay, which leads to redefining the traffic modeling and prediction problem with varying spatial context. Moreover, it is experimentally shown that sparse representation is valuable in detecting such varying spatial context in an adaptive manner, which outperforms the existing works with fixed spatial context in a nearby region as input.

### Data and Evaluation Metric

The data are continuously collected from thousands of loop detectors located on the Twin Cities Metro freeways by the Regional Transportation Management Center (RTMC), a division of Minnesota Department of Transportation, with a 30-second sampling rate (http://www.d.umn.edu/∼tkwon/TMCdata/TMCarchive.html). We use the data from 4 February 2012 to 14 March 2012, 40 days in total, to learn the weights in the sense of sparse representation for every sensor. The data of the subsequent 20 days, from 15 Match 2012 to 3 April 2012, are used to evaluate the prediction performance.

This dataset contains the data archived from thousands of sensors but some sensors do not work every day, that is, there are missing values. Besides, the data obtained from some sensors varies little from time to time. The data from such sensors are removed and only the data from 3254 sensors are preserved to test the proposed predictor. Prior to the test, we perform the preprocessing to accumulate the traffic volume per 10-minute interval for every sensor, which is a common practice for traffic volume prediction.

We employ the predicting accuracy defined in [14] as the metric for performance evaluation, that is,

$$\begin{array}{c} \;Accuracy=1-\frac{1}{n}\sum_{i=1}^{n}|\frac{v_{i}^{j}-{\bar{v}}_{i}^{j}}{v_{i}^{j}}|\times 100\%\; \end{array}$$ (11)

where *n* is the total number of the traffic volume values to be predicted,$v_{i}^{j}$ the actual traffic volume, and ${\bar{v}}_{i}^{j}$ the predicted one.

In the training process, we set the parameter *λ* = 0.001, which is the only parameter to be set as defined in Eq (9). We will discuss this in detail later.

### Temporal Correlation

**Fig 1:**
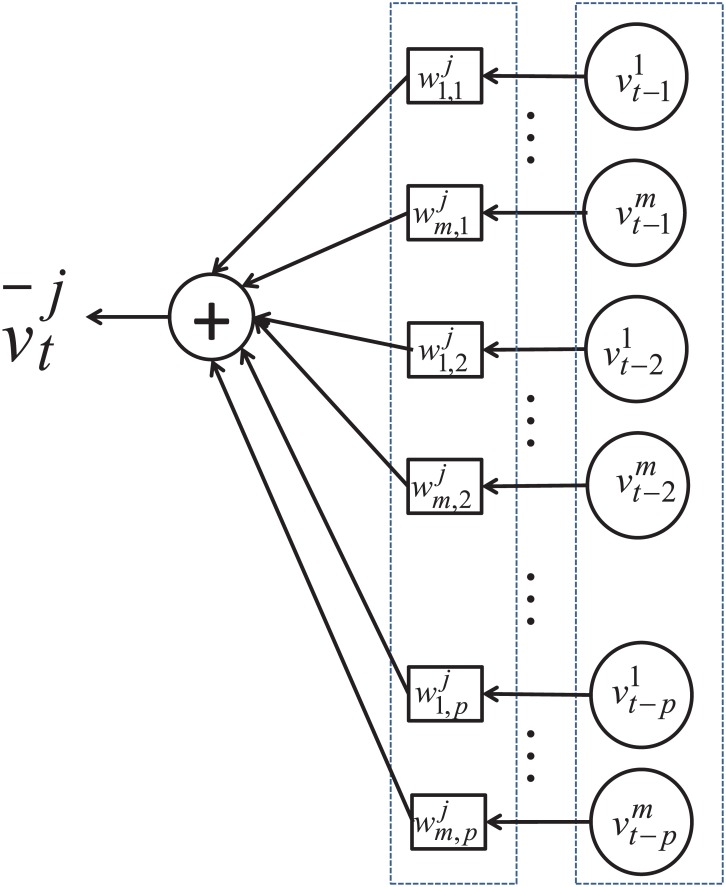
Predictor to infer temporal correlation.

Solving Eq (9) in the sense of sparse representation will result in a couple of nonzero elements in the weights defined in Eq (3) or Eq (4). Our concern is: Do such nonzero weights exist mainly in $W_{1}^{j}$ or distribute over $W_{1}^{j},W_{2}^{j},...,W_{p}^{j}$? If only $W_{1}^{j}$ contains nonzero weights, it will justify that the components of higher orders are redundant and contribute little to improve the prediction performance. The previous studies prefer high-order models [14][15]. The underlying assumption is: For the traffic flow at time *t* + *τ* that is being predicted at time *t*, the historical data in the time window [*t* − *p*Δ, *t*] are relevant and contributive to the prediction, where Δ is the time interval of 10 minutes in this study and *p* is the order of the predictive model in Eq (3). However, such an assumption of the time correlation is reasonable or not has never been checked. Our intuition is: To predict the traffic flows 10 minutes later, only the data at the present time are informative and the data of earlier times are not so relevant due to the evolution of the traffic flows. To verify this hypothesis, the prediction tests are conducted with *p* = 1, 2, …, 6 in Eq (3), respectively, which means that the traffic flow to appear after 10 minutes is predicted with the historical data of 10, 10 ∼ 20, 10 ∼ 30, 10 ∼ 40, 10 ∼ 50, and 10 ∼ 60 minutes in advance, respectively. The historical data of 10 ∼ 20 minutes in advance as input corresponds with such a case that the predictor of order *p* = 2 is applied, where the data sampled at *t*-10 minutes and *t*-20 minutes are combined to predict the traffic volume at time *t*. Similarly, the other scenarios can be defined with the order of the predictor up to *p* = 6, the input of which is the combination of the data sampled at 10, 20, 30, 40, 50, and 60 minutes in advance, respectively. The predictor of order *p* is illustrated in Fig 1.

The average accuracy for the 3254 sensors in the sense of Eq (11) is listed in Table 1, where the mean accuracies are 89.68%, 89.80%, 89.84%, 89.86%, 89.84%, and 89.85% for the range of the time interval for prediction to be 10, 10 ∼ 20, 10 ∼ 30, 10 ∼ 40, 10 ∼ 50, and 10 ∼ 60 minutes in advance, respectively. It is obvious that the performance improvement is minor when adding more components of higher orders in correspondence with the historical data of earlier than 10 minutes in advance. In Table 2, we present the results regarding how the nonzero variables distribute across different orders when considering the data in the time window of 10 ∼ 20, 10 ∼ 30, 10 ∼ 40, 10 ∼ 50, and 10 ∼ 60 minutes in advance as input, where the number of nonzero variables is the average number over the 3254 prediction tasks. It is apparent that whatever the time range of the input data is, most of the nonzero weights solved by sparse representation fall in the time range of the most recent 10 minutes, corresponding with *p* = 1. This reveals that the data obtained in the immediately previous step plays a more important role in traffic flow prediction than the data of a longer history.

**Table 1:** The mean accuracy against the time range of the historical data for predicting the traffic flows to appear after 10 minutes at 3254 sensors.

Time (min.)	10	10∼20	10∼30	10∼40	10∼50	10∼60
Accuracy (%)	89.68	89.8	89.84	89.86	89.84	89.85

**Table 2:** The average number of the nonzero weights of different orders for predicting the traffic flows to appear after 10 minutes at 3254 sensors.

	Number of nonzero weights of different orders					
Time range in advance(min.)	p = 1	p = 2	p = 3	p = 4	p = 5	p = 6
10 ∼ 20	100	59	0	0	0	0
10 ∼ 30	92	41	44	0	0	0
10 ∼ 40	88	36	33	35	0	0
10 ∼ 50	83	32	29	27	36	0
10 ∼ 60	81	30	26	23	28	34

Yet, it cannot be conclude from Tables 1 and 2 whether the high-order components in correspondence with *p* > 1 can be neglected in the predictor. In order to justify such issue, in the following, we conduct the prediction based on removing the components of the high orders of *p* > 1. Suppose that sensor *j* is the targeted one undergoing prediction and the indices of the 1-order nonzero components resulting from sparse representation as shown in Table 2 are noted as $R=\{i|w_{i,1}^{j}\neq 0\}$. We let the VAR predictor be composed by the nonzero 1-order components contained in R:

$$\begin{array}{c} \;{\bar{v}}_{t}^{j}=\sum_{i\in R}v_{t-1}^{i}w_{i,1}^{j}\; \end{array}$$ (12)

Then, least-square fitting is performed to tune the weights of the predictor defined in Eq (12). For the 5 cases with the time range of the input to be 10 ∼ 20, 10 ∼ 30, 10 ∼ 40, 10 ∼ 50, and 10 ∼ 60 minutes in advance, the mean accuracy of the 3254 sensors based on the 1-order predictive model is provided in Table 3. The accuracies in comparison with those in Table 1 are 89.44% against 89.80%, 89.40% against 89.84%, 89.36% against 89.86%, 89.31% against 89.84%, and 89.28% against 89.85%. It is obvious that the prediction performance based on the least squared fitting of the 1-order weights is almost as good as using all the sparse representation selected variables. So, it proves that temporal correlation over a longer history is a redundant part and 1-order prediction model is enough.

**Table 3:** The mean prediction accuracy of 3254 sensors based on only the nonzero variables corresponding with the p = 1 components in Table 2.

Time (min.)	10∼20	10∼30	10∼40	10∼50	10∼60
Accuracy (%)	89.44	89.4	89.36	89.31	89.28

We attribute the simple temporal correlation among traffic flows to the following view: The evolving of traffic flows over the whole road network can be regarded as the behavior of a dynamic system. With a bigger time lag, it is possible for vehicles to travel a longer distance to affect traffic flow distribution and variation in a wider area, and the state of the dynamic system may evolve into more possibilities correspondingly. This means that a longer history may bring in higher uncertainty in terms of prediction. That accounts for why a longer history of the traffic time series does not improve the prediction performance in comparison with the immediate state just one step ahead of the prediction. This also indicates that the traffic state of road network evolves in a mechanism like Markov chain since the present state is only relevant to the state of the last time.

In contrast to the simple temporal correlation, as a byproduct, it is notable that the minimum number of spatially relevant sensors solved from sparse representation is remarkably greater than what is assumed up to 25 sensors in accordance with Table 2, where the number of the relevant variables is close to 100, which coincides with the large scale correlations among traffic flows observed in [12]. Therefore, we will investigate into the spatial correlation in detail in the following.

### Spatial Correlation

**Fig 2:**
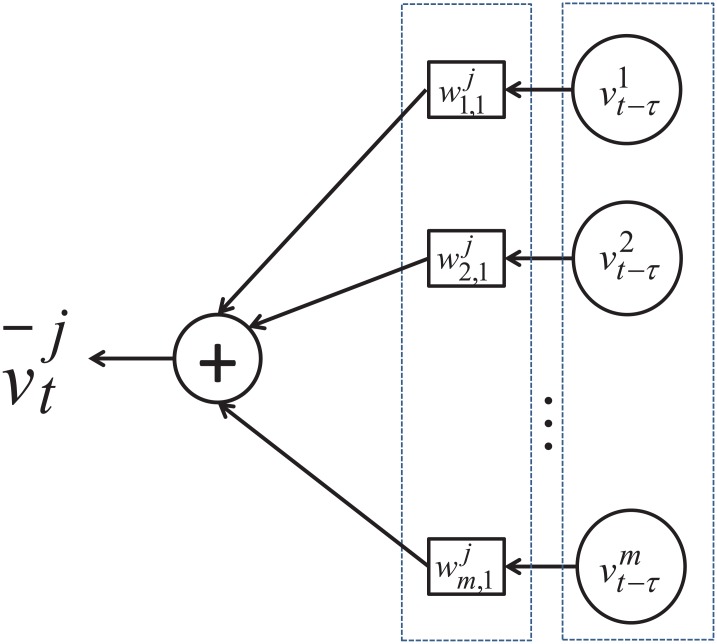
Predictor to infer spatial correlation.

According to the simple temporal correlation discovered as Markov chain, we can then focus on only the data of the last time so that the traffic flow evolution can be modeled as 1-order dynamic model for the sake of prediction as done in [24]. That is, remove the high-order components of *p* > 1 in Eq (3) to reduce the predictive model into 1-order model as follows: First, we redefine the notation of the traffic state of the whole network of *m* sensors at time *t* − *τ* as

$$\begin{array}{c} \;V_{\tau }={\left[v_{t-\tau }^{1},v_{t-\tau }^{2},...,v_{t-\tau }^{m}\right]}^{T}\; \end{array}$$ (13)

where $v_{t-\tau }^{i}$ represents the traffic volume observed at sensor *i* at time *t* − *τ*. Then, the goal is to fit the traffic state sequence *V* = [*V*_*τ*_,*V*_*τ*+1_,…,*V*_*τ*+*n*−1_]^*T*^ into the traffic time series at sensor *j*, say, $v^{j}={\left[v_{t}^{j},v_{t+1}^{j},\ldots ,v_{t+n-1}^{j}\right]}^{T}$, via the predictor defined as

$$\begin{array}{c} \;{\bar{v}}^{j}=Vw^{j}\; \end{array}$$ (14)

where $w^{j}={\left[w_{1,1}^{j},w_{2,1}^{j},\ldots ,w_{m,1}^{j}\right]}^{T}$ is a *m*-dimension vector functioning to weight the spatial correlations of all the *m* sensors in the road network with regard to the prediction task $V_{\tau }\Rightarrow v_{t}^{j}$ at sensor *j*. Here, ${\bar{v}}^{j}$ represent the predicted values of *v* ^*j*^. The predictor is illustrated in Fig 2. Eq (14) is also solved by using the LARS algorithm in the framework of sparse representation to optimize the performance index defined in Eq (9) so as to make the predicted values as close to the actual values as possible and in the meantime, the number of nonzero weights in *w* ^*j*^ as few as possible. Here, the nonzero elements in *w* ^*j*^ indicate the sensors correlated to the prediction task at sensor *j*, which are referred to as spatially correlated sensors, and the number of such nonzero variables is referred to as sparse number.

#### Large scale spatial context across the city

In this test, also, we fix the parameter value as *λ* = 0.001, and count the mean prediction accuracy in regard to the 3254 sensors. The traffic volume values to be predicted are from 7 AM to 8 PM on from 15 Match 2012 to 3 April 2012. We conduct the prediction with 10, 20, 30, 40, 50, and 60-minute time lag, respectively.

**Fig 3:**
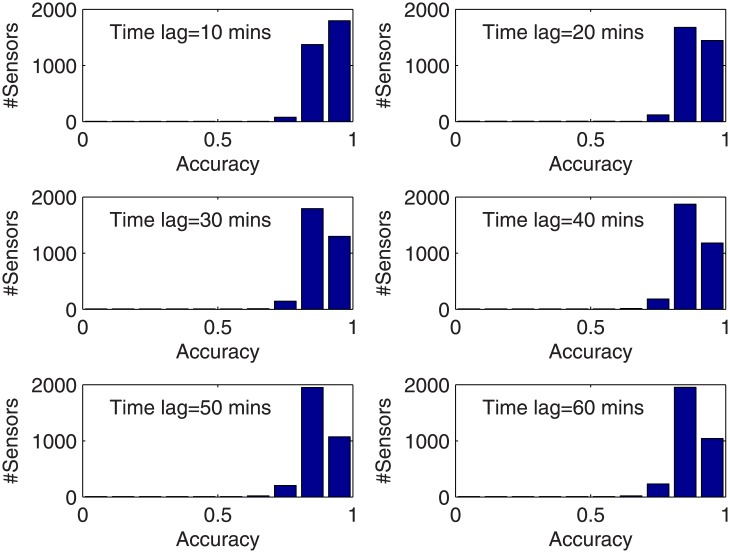
Distribution of the 3254 sensors on prediction accuracy.

**Fig 4:**
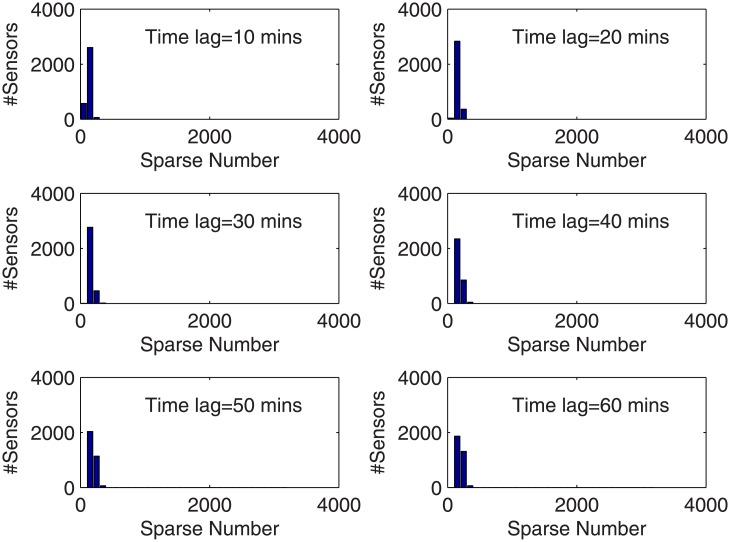
Distribution of the 3254 sensors on sparse number.

The prediction accuracies for sensor 1–10 and the averaged accuracy for the 3254 sensors in the sense of (11) are reported in Table 4. For the 6 cases with the time lag of 10-60 minutes, the mean accuracies are 89.68%, 88.46%, 87.96%, 87.45%, 87.02%, and 86.85%, respectively. Table 4 also lists the sparse number obtained for sensor 1–10 and the mean sparse number for the 3254 sensors. The histograms regarding the distribution of the 3254 sensors on different ranges of prediction accuracy as well as sparse number are provided in Figs 3 and 4, respectively. It is obvious that the prediction accuracy is between 0.8 and 0.9 in most cases, and even higher. Only in a few cases, the prediction accuracy is between 0.7 and 0.8. For the sparse number, the dominant case is 100-200.

**Table 4:** The prediction accuracy and the number of sparse representation selected variables for 10 sensors and the overall performance of 3254 sensors under different time lags.

		Time lag					
Sensor		10 min.	20 min.	30 min.	40 min.	50 min.	60 min.
Accuracy(%)	1	87.8	85.68	84.57	84.4	83.73	84.45
	2	91.54	90.64	90.4	90.16	89.95	90.19
	3	87.23	83.9	81.8	82.2	82.23	82.11
	4	87.28	84.35	82.11	82.24	82.42	81.96
	5	88.18	85.16	82.98	83.28	83.12	82.56
	6	88.19	84.86	82.95	83.05	83.39	82.72
	7	86.22	84.71	84.67	84.77	84.17	83.47
	8	93.14	92.09	91.65	91.39	91.01	91.1
	9	93.41	91.96	91.34	90.97	90.58	90.12
	10	87.62	85.44	83.85	82.67	83.18	83.23
	All	**89.68**	**88.46**	**87.96**	**87.45**	**87.02**	**86.85**
Sparse number	1	117	156	171	186	225	214
	2	115	156	153	158	180	172
	3	173	210	219	240	265	235
	4	157	211	220	245	261	243
	5	156	215	226	242	241	243
	6	159	211	223	241	238	251
	7	143	196	187	189	231	208
	8	121	137	153	158	174	184
	9	108	125	157	172	182	191
	10	136	187	196	232	230	230
	All	**125**	**155**	**165**	**183**	**196**	**202**

**Fig 5:**
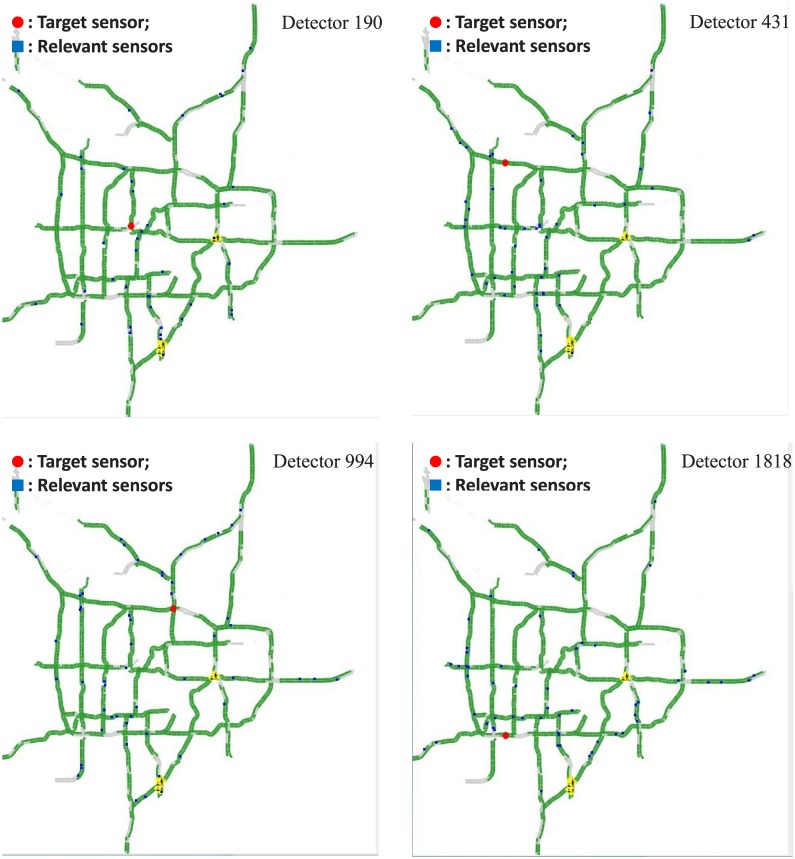
Spatial contexts for given prediciton tasks.

We see 3 important points from Table 4, Figs 3 and 4: First, the sensors contributive to a prediction task distribute widely in the road network, in general over 100 in order to achieve high prediction performance. This coincides with the results obtained in [12]. It indicates that spatial correlations exist in a much wider range than what was assumed previously. To justify this, a few examples of the spatially relevant sensors solved from sparse representation are illustrated in Fig 5. We can see that the relevant sensors distribute in a wide range of the whole road network. It is interesting to notice that in every case in Fig 5, the detectors located in the main entrances of the road network are selected as relevant sensors. This means that the overall traffic flows enter and leave a city should have significant impact on the forming of the traffic patterns in the city. To further investigate into whether the global spatial contexts follow a general law or represent just a few exceptional examples, we list in Table 4 the average number of the spatially relevant sensors for prediction under different time lags, which shows that the majority of the sparse number is over 100 under the time lag for prediction from 10 to 60 minutes. To allow a view with more details, the distribution of the sparse number for the 3254 prediction tasks is illustrated in Fig 4. Second, according to Table 4 and Fig 4, the number of spatially correlated sensors with regard to a prediction task is subject to which sensor is undergoing prediction, varying from case to case. Third, the subset of variables/sensors relevant to a prediction task can be determined automatically using the sparse representation based methodology. The above observations coincide with the facts observed in [12]. Another fact observed in Table 4 is that in general, the sparse number increases with the time lag for prediction.

We explain it as follows: With a bigger time lag, it is possible for vehicles to travel a longer distance to affect the traffic flow distribution and variation in a wider area, and the traffic state of the whole network can thus evolve into more possibilities. This accounts for why the number of spatially correlated sensors increases while the predictability decreases with the increment of the time lag.

#### Comparison with unselective global context for prediction

Although we observe a large scale correlation among traffic flows for a prediction task based on sparse representation and the accuracy reported in Table 4 is as high as 89.68% for the prediction with the time lag of 10 minutes, it is necessary to check whether the high performance does really result from sparse representation. Therefore, we compare the proposed method with the least squared fitting method, which is a predictor based on using the data from all sensors. The results listed in Table 5 show that the prediction performance obtained by using sparse representation is much better than using the data from all sensors, that is, 89.68% against 81.83%, which is achieved by applying least square fitting to solve Eq (14).

**Table 5:** Comparison with the least squared fitting method in terms of 10-minute-ahead prediction accuracy (%).

Sensor	LS	Sparse
1	76.32	87.8
2	84.36	91.54
3	78.18	87.23
4	79.38	87.28
5	80.11	88.18
6	80.32	88.19
7	75.57	86.22
8	85.34	93.14
9	88.51	93.41
10	79.86	87.62
All	**81.83**	**89.68**

#### Comparison with neighborhood based spatial context for prediction

Traditionally, spatial correlations are granted as a couple of neighboring sensors of a fixed number around the target sensor under prediction. The experimental results obtained in this study lead to an opposite point in two aspects: (1) Spatial correlations exist in a much wider area than just a couple of nearest neighbors. Prediction based on such wide-area correlations in the framework of sparse representation outperforms the prediction the spatial contexts of which refer to just a couple of sensors in the neighborhood. (2) The number of sensors relevant to a prediction task should not be a fixed number but subject to the sensor undergoing prediction as well as the time lag, which can be determined automatically via the so-called sparse representation technique. To verify these, we compare the performance of the sparse representation based solution with that of neighborhood based prediction using 4 predictors, namely linear model, back propagation (BP) neural network, radical basis function (RBF) neural network, and vector autoregressive (VAR) model. In Table 6, the mean prediction accuracy over 60 sensors is listed for each predictor with time lag from 10 to 60 minutes. For each row in Table 6, the same predictor is applied but the spatial context is defined as 15, 20, 25, 30 neighboring sensors, and that determined automatically via sparse representation so as to evaluate whether sparse representation is a more reasonable scheme in revealing the spatial causal for traffic volume prediction. As highlighted in Table 6, prediction based on sparse representation leads to the highest mean accuracy in almost all cases no matter which predictor and what time lag are applied. This shows that sparse representation is a more reasonable methodology in detecting spatial correlations for traffic volume prediction as it leads to higher accuracy and less performance variation.

**Table 6:** Mean prediciton accuracy of 60 sensors under different time lags with input from 10, 15, 20, 25, 30 neighboring sensors, and sparse representation (%).

		Number of sensors (input)					
	Time lag	10	15	20	25	30	Sparse
Linear	10 min.	87.9	88.25	88.52	88.68	88.83	**91.23**
	20 min.	86.46	85.93	86.15	86.29	87	**89.95**
	30 min.	84.98	84.18	84.46	84.62	85.56	**89.37**
	40 min.	83.23	82.14	82.46	82.69	83.91	**88.85**
	50 min.	81.77	80.24	80.66	80.95	82.58	**88.38**
	60 min.	80.43	78.36	78.88	79.25	81.33	**88.27**
BP	10 min.	89.19	89.43	89.56	89.63	89.76	**89.83**
	20 min.	87.7	87.9	88.01	88.08	**88.25**	87.94
	30 min.	86.66	87.05	87.14	87.31	**87.53**	87.07
	40 min.	85.4	85.88	85.99	86.26	86.47	**86.64**
	50 min.	84.5	84.91	85.18	85.51	85.71	**86.41**
	60 min.	83.53	84.25	84.59	84.99	85.34	**86.32**
RBF	10 min.	88.94	89.24	89.41	89.43	89.48	**91.17**
	20 min.	87.49	87.75	87.78	87.65	87.78	**89.76**
	30 min.	86.44	86.81	86.89	86.84	86.98	**89.2**
	40 min.	85.08	85.54	85.67	85.75	85.83	**88.83**
	50 min.	84.06	84.6	84.78	84.92	84.98	**88.55**
	60 min.	83.13	83.79	84.04	84.27	84.38	**88.74**
VAR	10 min.	89.46	89.58	89.69	89.76	89.87	**91.14**
	20 min.	87.35	87.47	87.59	87.64	87.76	**89.62**
	30 min.	85.73	85.83	85.98	86.06	86.2	**89.01**
	40 min.	83.72	83.85	84.05	84.14	84.28	**88.75**
	50 min.	81.97	82.09	82.32	82.39	82.52	**88.4**
	60 min.	80.25	80.4	80.67	80.75	80.88	**88.27**

**Fig 6:**
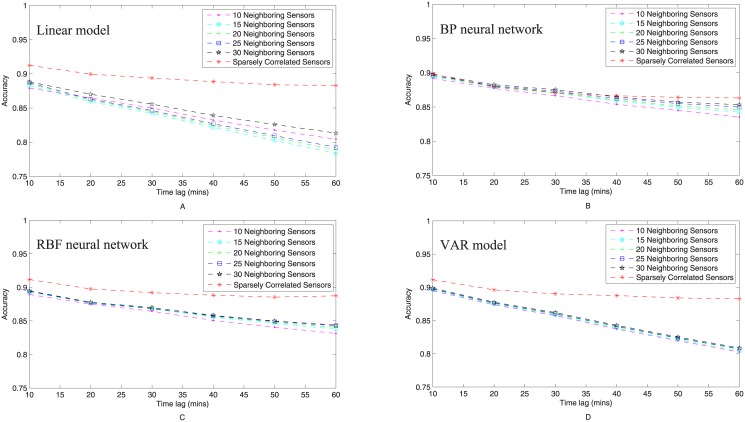
Performance degradation of each model with increment of time lag for traffic volume prediction.

If we pay attention to each column of Table 6, we can find that the performance degrades with the increment of time lag for the prediction based on any spatial context but this is more severe for the cases of neighborhood based spatial contexts. To enable a more straightforward comparison, we illustrated in Fig 6 the decay of prediction performance against time lag for every spatial context applied in the comparative study. It is apparent that the performance degradation of sparse representation selected spatial context is not obvious accompanying the increment of time lag but that of neighborhood based spatial context is remarkably notable. Such a phenomenon is explained as follows: When time lag increases, almost all vehicles on the road network can travel a longer distance to affect the traffic conditions in a broader area, which accounts for why spatial correlations exist in a wider area and enlarge with time lag. If return to Table 4, we can see that the spatial context (the number of relevant sensors) enlarges with the increment of time lag for sparse representation based solution. As the neighborhood based predictors force spatial correlations to lie in a couple of fixed neighboring sensors, they cannot adapt to the changing spatial correlations subject to time lag, which deviates from the practice. In contrast, as a fully automated method, the relevant sensors selected by sparse representation do reflect such change of spatial correlations. This justifies from another perspective the reasonableness of applying sparse representation for spatial context mining while evidences the point that traffic flow prediction should not be simply a task of monitoring and foreseeing how nearby traffic flows propagate but a more complex task involving city dynamics at a more extended scale. Note that BP neural network performs always the worst in all experimental settings. It is known that the output of BP neural networks is the weighted sum of the outputs of the neurons in the hidden layer, which are actually the mixture of the input variables. In another word, the inputs variables are mixed again in the hidden layer and then undergo nonlinear transformation, so there are no independent input variables in the outputs of the hidden layer. In such a case, variable selection does not work for BP neural networks, so the performance is relatively low. This also explains why sparse representation does not outperform neighborhood based prediction in all the cases when applying BP neural networks as the predictor.

### Parameter Setting

It is apparent that Eq (9) will penalize *w* ^*j*^ when it has big ${\left\|w^{j}\right\|}_{1}$ or when it leads to big mean-square-root error *ɛ*_0_ in correspondence with ${\left\|Vw^{j}-v^{j}\right\|}_{2}^{2}$. So, Eq (9) requires both the sparseness of *w* ^*j*^ and the minimized fitting error *ɛ*_0_. The Lagrange multiplier *λ* controls the balance of the penalty to both terms. If *λ* is big, Eq (9) will penalize ${\left\|w^{j}\right\|}_{1}$ more than *ɛ*_0_. Otherwise, it will penalize *ɛ*_0_ more. As a result, big *λ* will produce a sparse solution but such a solution may not fit well into historic data while small *λ* will lead to a solution fitting well into historic data but possibly not being so sparse. Therefore, *λ* is positively relative to *ɛ*_0_ and negatively relative to ${\left\|w^{j}\right\|}_{1}$.

**Fig 7:**
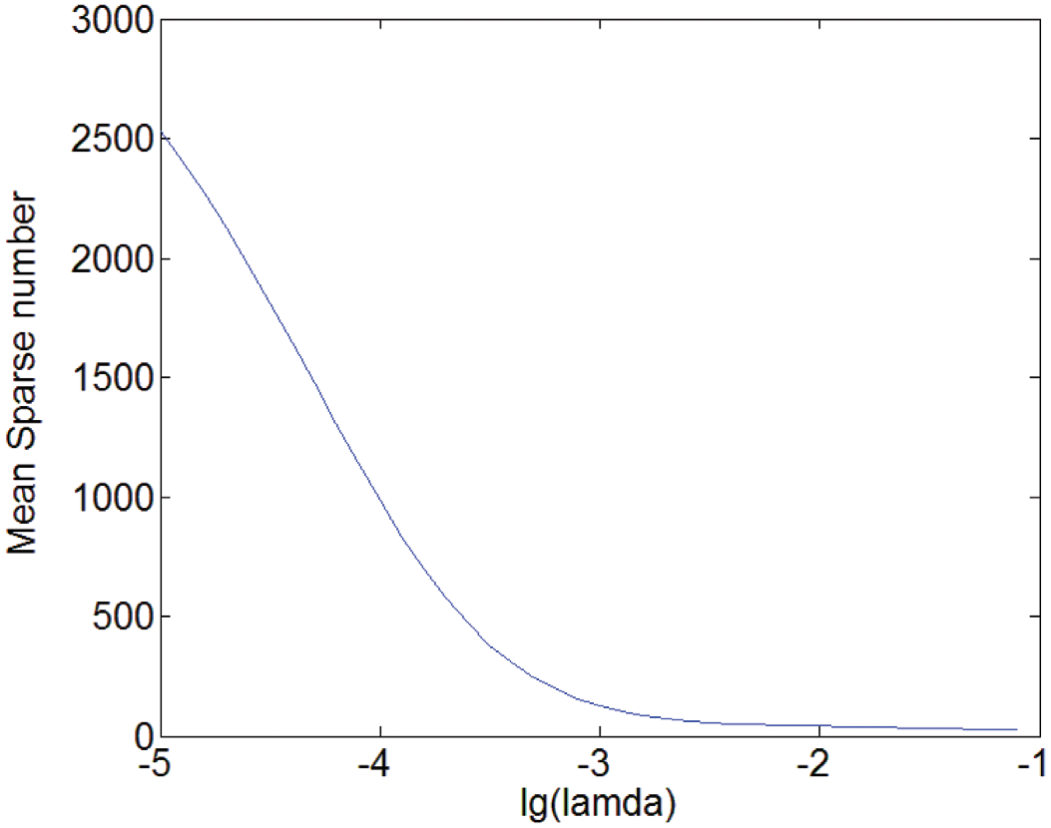
Mean sparse number against lg(*λ*).

**Fig 8:**
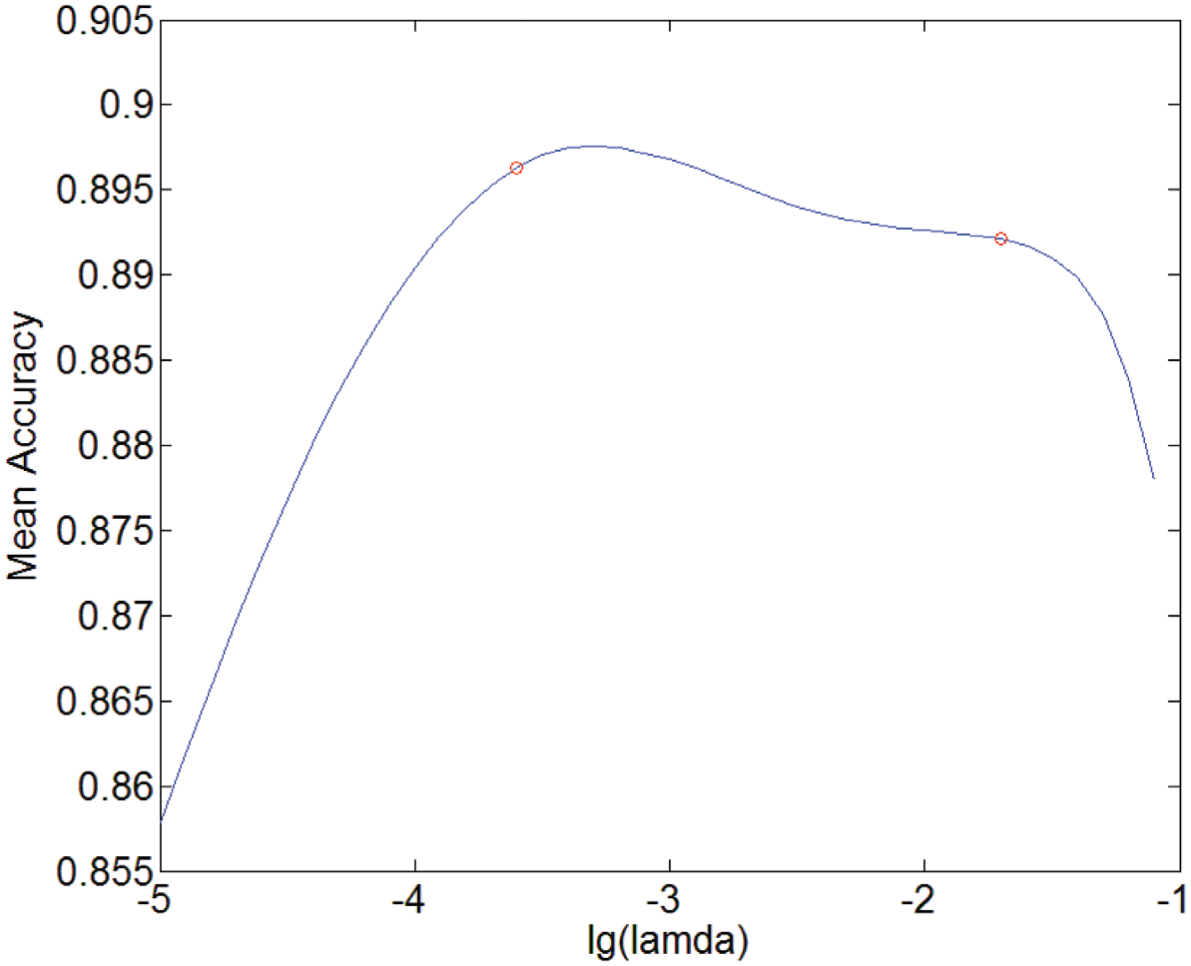
Mean accuracy against lg(*λ*).

In Fig 7, we illustrate the relation between mean sparse number and *λ* and in Fig 8, that between the mean prediction accuracy and *λ* for the prediction tasks at 3254 sensors. Note that since *λ* is so small that falls in the range [0.00001, 0.1], we apply ${\text{log}}_{10}^{\lambda }$ in Figs 7 and 8. It can be seen that there exists a stable range in which the prediction accuracy remains high and varies little, where *λ* is nearly from 0.0003 to 0.02.

**Fig 9:**
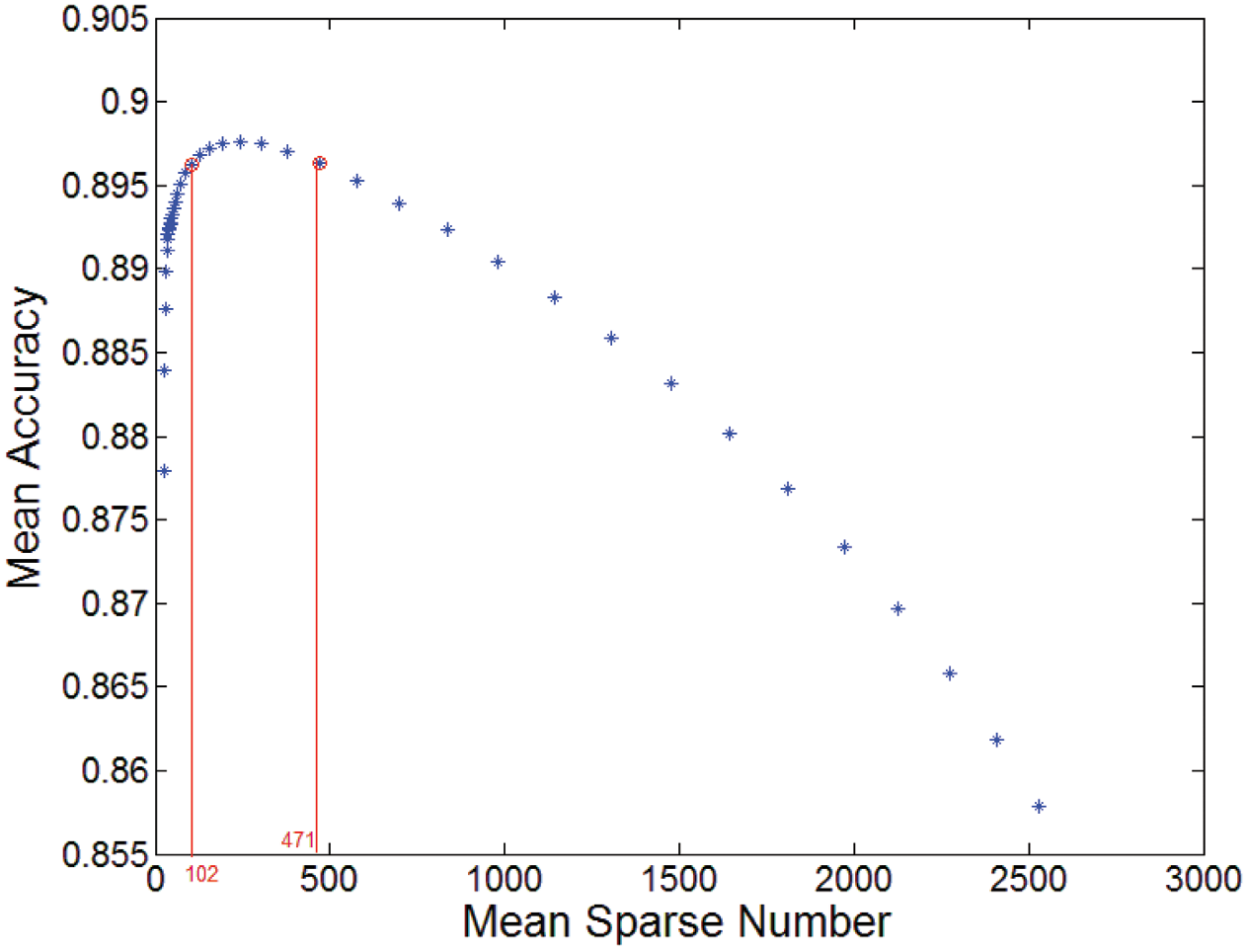
Mean accuracy against mean sparse number.

It is known that every possible value of *λ* can determine a pair of values for sparse number and accuracy. In Fig 9, we figure out the relationship between the mean sparse number and the mean accuracy over the 3254 sensors. From this figure, we see that the sparse number leading to high accuracy with little fluctuation is approximately within [100, 500]. The accuracy is low when the sparse number is too small and it drops drastically when the sparse number is less than 100. In such a case, since the relevant sensors included in the predictive model are inadequate, the predictive performance is for certain not good. So, enough number of spatially correlated sensors should be included in the predictive model and 100 is the rough boundary as observed in the experiments. This implies that spatial correlations should exist widely on the road network of interest in terms of predicting the traffic volume at a given sensor. On the other hand, when the sparse number is too big, the accuracy is also low. The accuracy decreases obviously when the sparse number is nearly over 500. We attribute this phenomenon to the following point of view: As the sparse number exceeds a certain number, roughly 100 in this study, more spatially correlated sensors are included in the predictor but the information from such sensors could be redundant. In such a case, the accuracy does not increase with the increment of the number of relevant sensors. When the number of the sensors applied in the predictor increases continuously to exceed a up bound, 500 in this study, increasingly more irrelevant sensors will be included, which will degrade the prediction performance. These account for why the accuracy decreases so fast as the sparse number becomes smaller than 100 while it decreases slowly when the sparse number is greater than 500. The above observations show that the spatial correlations in term of predicting traffic volume at a given sensor are much wider than what was assumed previously. Here, the range of the number of spatially correlated sensors should be within [100, 500].

In the tests of this study, we fix the parameter value as *λ* = 0.001, which falls in the stable range (0.0003, 0.02).

### Discussions

In sum, some interesting phenomena are observed in the experiments: (1) Prediction based on the sparse representation selected sensors outperforms that based on using all sensors. Besides, it outperforms forcing the spatial context to lie in a certain range of neighborhood only. (2) Spatial correlations for traffic flow prediction exist in hundreds of sensors distributed on the whole road network sparsely, not just the neighborhood around the sensor of interest as assumed previously. (3) The spatial context relevant to a prediction task varies with the sensor of interest as well as time lag, so confining spatial correlations to a fixed number of neighboring sensors should not comply with the practice. Furthermore, these involve the understanding of how traffic flows are formed from human mobility. It is discovered that most people in a city travel to a few determined destinations with limited routing choices from day to day [31]. So, prediction based on global configuration of traffic flows is possible in that since the route of every individual is mostly fixed and the traffic volume is the assembly of all such individual routing, the signals at far ends could indicate the formation of the traffic flows nearby. Fig 5 may provide some clues: The sensors at some entrances of the road network are usually detected as relevant sensors and the high traffic flow at a given entrance may affect the traffic flows at some certain links of the road network in the near future provided the traveling route of each user remains almost fixed. Moreover, people are in general able to travel to farer places in a longer time so as to affect the traffic flows in a larger area, so the spatial context is not fixed but subject to time lag. When the time lag for prediction increases, the automated variable selection rendered by sparse representation can adapt to the larger spatial context but the neighborhood based methods do not, this accounts for why the prediction accuracy of sparse representation decreases much more slowly than that of neighborhood based methods.

## Conclusion

Spatiotemporal context mining from city-scale big data should be a timely issue for traffic flow prediction due to the superior performance in contrast to the local context based prediction, but remains a missing topic so far. The primary contribution of this study is: A new framework is proposed to solve the traffic flow prediction problem from a big data point of view in terms of city-scale spatial-temporal correlation mining. In detail, this study contributes in two aspects: (1) Spatial-temporal context mining is formulated as a variable selection problem to optimize regression based traffic flow prediction; (2) Sparse representation is proposed to solve the spatial-temporal context mining problem in terms of variable selection. Here, sparse representation is applied as a variable selection method to discover spatial-temporal correlations among the traffic data at the whole city scale for traffic flow prediction, which makes it different from all the existing predictors focused on local spatial contexts. To the best of our knowledge, it is the first investigation to introduce the sparse representation based solution for traffic flow prediction with city-scale signatures.

Another notable contribution of this study is the experimental findings. Due to the proposal of utilizing sparse representation for spatial context mining, we observe some phenomena that have never been found before: (1) Temporal correlation is very simple that can be represented as 1-order dynamic model. (2) The sensors relevant to a prediction task distribute all over the city, not just in a local region. The spatial context can spread out far away to the edge of a city with over 100 relevant sensors distributing widely in the whole city. (3) The number of relevant sensors is not a fixed concept but varies with the target sensor undergoing prediction as well as the time lag for prediction. In fact, the spatial context enlarges with the increment of time lag because a longer travel time allows travelers to go through a longer distance to affect the traffic flows in a larger area. Sparse representation-inferred relevant sensors can reflect such changing spatial context adaptively and as a consequence, it promises better prediction accuracy in comparison with the traditional models focused on neighborhood based fixed spatial context for prediction.

The aforementioned experimental findings are meaningful in that they call for reconsidering the widely adopted practice to enforce the spatial context for traffic flow prediction to lie in a neighborhood with fixed number of sensors as input subjectively and manually. Moreover, such experimental findings as well as the thought of rendering traffic flow prediction in the framework of big data analytics (city-scale spatial context mining) should be informative when developing new predictors.
